# Increasing the horizontal orientation of transition dipole moments in solution processed small molecular emitters†
†Electronic supplementary information (ESI) available: Synthesis of *t*Bu-DACT-II; optical constants of TPBi and PFO; fluorescence emission of PFO, PFO:OPV7 (2%) and TPBi:OPV7 (2%); AFM height scan of PFO:OPV7 (2%), not annealed and annealed layers; angular dependence of *p*-polarised photoluminescence of TPBi:Ir(MDQ)_2_(acac) evaporated layer (graph and table with value); description of OrientExpress. See DOI: 10.1039/c7tc01568b


**DOI:** 10.1039/c7tc01568b

**Published:** 2017-06-12

**Authors:** Alessia Senes, Stefan C. J. Meskers, Horst Greiner, Katsuaki Suzuki, Hironori Kaji, Chihaya Adachi, Joanne S. Wilson, René A. J. Janssen

**Affiliations:** a Holst Centre/TNO , High Tech Campus 31 , P.O. Box 8550 , 5605 KN Eindhoven , The Netherlands . Email: alessia.senes@tno.nl; b Molecular Materials and Nanosystems and Institute for Complex Molecular Systems , Eindhoven University of Technology , P.O. Box 513 , 5600 MB Eindhoven , The Netherlands . Email: r.a.j.janssen@tue.nl; c Consultant , . Email: horst.greiner@netaachen.de; d Institute for Chemical Research , Kyoto University , Uji , Kyoto 611-0011 , Japan . Email: kaji@scl.kyoto-u.ac.jp; e JST , ERATO , Adachi Molecular Exciton Engineering Project , 744 Motooka , Nishi , Fukuoka, 819-0395 , Japan . Email: adachi@cstf.kyushu-u.ac.jp; f Center for Organic Photonics and Electronics Research (OPERA) , Kyushu University , 744 Motooka, Nishi , Fukuoka 819-0395 , Japan . Email: adachi@opera.kyushu-u.ac.jp

## Abstract

The efficiency of organic light emitting diodes (OLEDs) can be improved by controlling the orientation of the transition dipole moment of the emitters. Currently, no effective methods exist for orienting the transition dipole moments in solution processed active layers for OLEDs. We investigate the orientation of the transition dipole moment of small molecular emitters in a host matrix of poly(9,9-dioctylfluorene) (PFO) by means of angle dependent luminescence intensity measurements. The polymer chains of the host orient predominantly in the plane of the film. Fluorescent *p*-phenylenevinylene oligomers with 6 and 7 repeat units (OPV6, OPV7) are found to also orient preferentially horizontally. The orientation of the emitters can be improved by thermal annealing with up to 90% of transition dipole moments oriented in the plane of the film. The phosphorescent emitter Ir(MDQ)_2_(acac) shows a degree of horizontal orientation in the polymeric host matrix lower than that which is observed for oligomers, but as high as is observed for the same emitter in evaporated layers. A carbazole derivative capable of thermally activated delayed fluorescence shows a small preference for vertical orientation within the polymer host. The strong orientation of OPV6 and OPV7 in the oriented polymer host is rationalized in terms of their high aspect ratios. The use of PFO as host material in host/guest systems allows achieving horizontal orientation of transition dipole moments in solution processed oligomers and small molecular emitters.

## Abstract 2

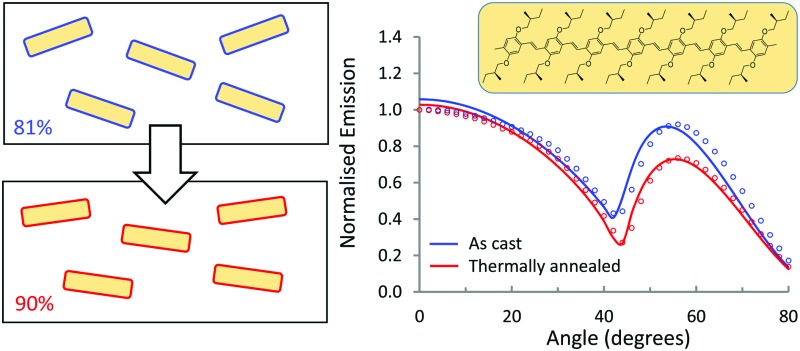
The transition dipole moments of the small molecular emitters in a polyfluorene host matrix align strongly in the plane of the film, when deposited from solution and even more when thermally annealed.

## Introduction

In recent years, OLEDs have gained a sizable share of the market of flat panel displays and are applied in smartphones and other portable electronics. Attractive features of OLEDs are large area, low weight, patternability and flexibility. On the lab scale, energy conversion efficiencies up to 139 lm W^–1^ for OLEDs that emit white light, have been achieved.^1^ While the internal quantum efficiency (IQE) of OLEDs involving phosphorescent molecular emitters is usually close to 100%, the external quantum efficiency (EQE) of the OLEDs is limited by the outcoupling efficiency of the light that has been generated within the emitter layer. The outcoupling efficiency rarely exceeds 20%. An important loss mechanism is waveguiding of light in the plane of the diode.^2^ An elegant way to improve the light outcoupling efficiency is to optimize the orientation of the transition dipole moment of the emitters.^3^–^6^ Optical analysis indicates that OLEDs with an EQE above 40% can be realized without any extra light extraction layer, if the ratio of horizontal to vertical components of the emitting transition dipoles exceeds 95%.^7^ Using small molecular emitters that show either phosphorescence or thermally activated delayed fluorescence (TADF), which have been deposited in a way which gives them mainly horizontal orientation in the active layer, diodes with EQE higher than 30% have been realized.^8^–^12^

Preferential orientation of the transition dipole moment of small molecular emitters in the horizontal plane of the organic layer for OLEDs has currently only been observed for layers deposited by thermal evaporation in high vacuum.^6^,^13^ However, a significant research effort is currently devoted to deposition of the organic layers from solution, which would allow for roll-to-roll processing, opening a way to high throughput and lower production cost. Lampe *et al.*^14^ compared the preferential orientation of iridium-based emitters in various host matrices deposited by solution processing and by thermal evaporation. They observed that only the evaporated layers show preferential horizontal orientation of the iridium emitters and concluded that horizontal orientation in the films of common phosphorescent iridium complexes cannot be achieved by solution processing. The preferential horizontal alignment in the deposition from the gas phase has been related to the presence of an interface between the aromatic matrix and vacuum during the deposition process.^15^

Preferential horizontal molecular orientation in solution processed layers is known for luminescent π-conjugated polymers.^16^–^19^ High degrees of horizontal orientation have also been recently reported for light-emitting oligomers in solution processed host/guest systems.^20^–^22^

**Fig. 1:**
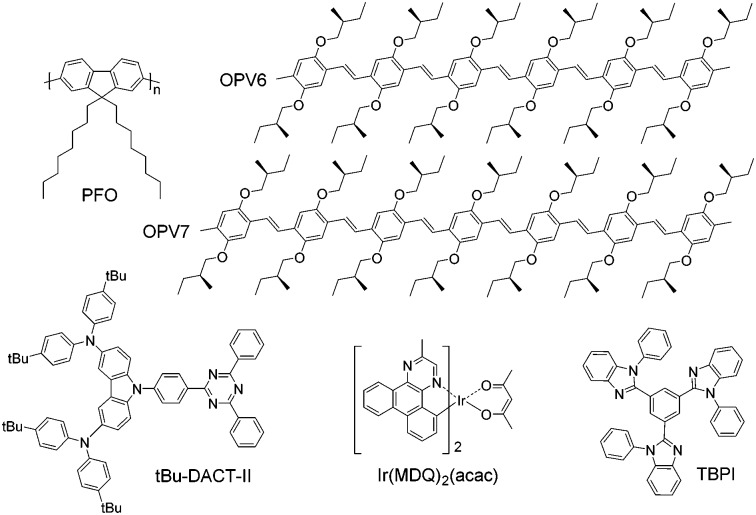
Chemical structures of host and guest molecules used in this work. Host materials: TPBi and PFO. Guest molecules: OPV6, OPV7, Ir(MDQ)_2_(acac) and *t*Bu-DACT-II.

In this work, we investigate the use of a π-conjugated polyfluorene host polymer deposited from solution for orienting molecular emitters embedded in the host. We use poly(9,9-dioctylfluorene) (PFO, see Fig. 1). For films of PFO deposited from solution by spin coating, preferential horizontal orientation of the polymer chains has been reported.^23^ We investigate four, rather different, molecular emitters which are each embedded in the polymeric host (Fig. 1) *via* solution processing to determine if PFO has the capability to orient the transition dipole moment of small molecular emitters. The guests represent characteristic fluorescent and phosphorescent molecular emitters with relevance for OLED applications. They possess very different shapes and the comparison between them enables assessing the influence of the aspect ratio of the molecules on the orientation of their transition dipole moment in solution processed films. The set of emitters includes two π-conjugated methyl end-capped *p*-phenylenevinylene oligomers containing either 6 or 7 phenyl rings (OPV6 and OPV7). These oligomers have a high structural aspect ratio and their transition dipole moment is oriented along the main chain.^24^ We have previously shown that at high concentration (10 wt%) OPV6 and OPV7 are horizontally aligned in solution processed layers of 2,2′,2′′-(benzene-1,3,5-triyl)tris(1-phenyl-1*H*-benzimidazole) (TPBi) as host, where they aggregate.^20^ In solution-processed^20^ and evaporated^25^ pristine layers, TPBi is oriented randomly. By using a polymeric host such as PFO that is able to align horizontally by itself we hope to induce horizontal alignment of the OPV6 and OPV7 guests in solution-processed layers at low concentration where aggregation is absent.

Next to OPV6 and OPV7, we investigate the red phosphorescent emitter iridium(iii)bis(2-methyldibenzo-[*f*,*h*]quinoxaline)(acetylacetonate) (Ir(MDQ)_2_(acac)). In evaporated host–guest layers Ir(MDQ)_2_(acac) shows preferential horizontal orientation,^5^,^15^,^25^–^28^ Quantum chemical calculations have shown that the horizontal orientation originates from the preferred direction of the triplet transition dipole moment of Ir(MDQ)_2_(acac) within the co-host environment.^28^ To the best of our knowledge such alignment has not been observed for solution-processed layers of iridium-based emitters,^14^ but with the self-aligning PFO host this may be accomplished.

Finally, we use also a highly emissive TADF small molecule, *t*Bu-DACT-II, which is a solution processable version of 9-[4-(4,6-diphenyl-1,3,5-triazin-2-yl)phenyl]-*N*,*N*,*N*′,*N*′-tetraphenyl-9*H*-carbazole-3,6-diamine (DACT-II).^8^ TADF emitters receive considerable attention in recent years as a promising new generation of emitters for OLED application,^29^ and being able to align TADF emitters horizontally in a suitable solution processed host matrix would further enhance their applicability for highly efficient OLEDS.

In the following we demonstrate that a high structural aspect ratio is necessary to achieve highly oriented transition dipoles in thin films. The OPV guest molecules, adopt predominantly an in-plane orientation when processed together with the polymer host from solution. Also for Ir(MDQ)_2_(acac) a partial horizontal alignment is established, while for *t*Bu-DACT-II preferential vertical alignment occurs, in accordance with the fact that the transition dipole moment and long axis of this molecule may not coincide.

## Experimental

### Materials and thin films

TPBi and Ir(MDQ)_2_(acac) were obtained from Lumtec and PFO from American Dye Source, Inc. The synthesis of the OPV6 and OPV7 molecules has been described previously.^30^*t*Bu-DACT-II was synthesized according to Scheme S1 (ESI†). For the PFO:OPV6 (5% by weight) and PFO:OPV7 (5%) blends, the two components of the host/guest system were mixed in a 19 : 1 weight ratio, and for the blend PFO:OPV7 (2%) in a 49 : 1 ratio; the materials were all dissolved in toluene, 0.5 w/v%, and deposited by spin coating at a speed of 800 rpm on quartz substrates, giving layers with thickness around 30 nm. For the PFO:Ir(MDQ)_2_(acac) blend, the two components of the host/guest system were mixed in a 9 : 1 weight ratio; they were dissolved in toluene, 1 w/v%, and deposited by spin coating at a speed of 1800 rpm on quartz substrates, resulting in 56 nm thick layers. For the TPBi:Ir(MDQ)_2_(acac) system, the components were dissolved in toluene, 0.5 w/v%, in a 9 : 1 ratio, and deposited on a substrate by spin coating at 800 rpm (20 nm thick layers). For the blend PFO:*t*Bu-DACT-II, the two components were mixed in a 91 : 9 weight ratio, dissolved in toluene 0.5 w/v%, and deposited by spin coating at a speed of 800 rpm on quartz substrates. These layers were 35 nm thick. In the blend TPBi:*t*Bu-DACT-II, the blending ratio was 91 : 9, the solution was dissolved in toluene, 1 w/v%, and deposited on quartz substrate at 1800 rpm, for 40 nm thick layers. Finally, PFO, 1 w/v% solution, was spin coated at 1800 rpm, giving 57 nm thick layers. All solutions were stirred at 70 °C for a few hours, allowed to cool down and then filtered through a 0.22 μm PTFE filter before deposition by spin coating on clean and UV-ozone treated quartz substrates. Layer thickness was determined by surface profilometry. The layers referred as “annealed” were subjected to temperature treatment for 10 minutes at 100 °C. All measurements, however, were done after cooling of the sample to room temperature. Preparation of solutions and films were performed in inert N_2_ atmosphere (H_2_O and O_2_ < 1 ppm).

### Angular dependent fluorescence

**Fig. 2:**
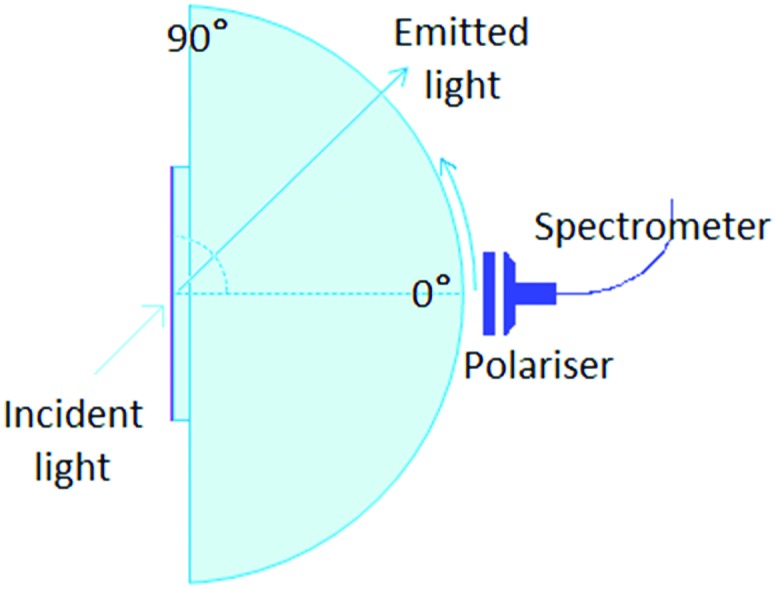
Layout of the experimental setup used for the angle-dependent fluorescence measurement. The organic layer used for the angle dependent luminescence measurements is on top of a quartz substrate, in optical contact with a quartz hemicylinder through refractive index matching oil. The organic layer (on the free side of the substrate) is excited using a UV LED with an incident angle of 45°. The fluorescence is collected through a polarizer and a fibre-coupled spectrometer.

The angular dependence of the *p*-polarised fluorescence intensity was determined experimentally in order to deduce the orientation of the transition dipole moment of the emitters embedded in the solution processed layers. The setup used (Fig. 2) was assembled in our laboratories based on the similar setup by Brütting *et al.*^31^ The layers are excited with the focused light of a 365 nm LED, at a fixed incident angle of 45°; the fluorescent light emitted through the quartz substrate, which is optically coupled to a quartz hemicylinder through matched refractive index oil, is *p*-polarised through a polarizer and collected with a spectrometer. The collected light is filtered with a 450 nm high pass filter, to avoid saturation. The fluorescence is measured at different angles, from 0° to 80°, in steps of 2°. The measurements are performed in inert N_2_ atmosphere (H_2_O and O_2_ < 1 ppm), to avoid photochemical degradation and photoluminescence quenching induced by combination of oxygen and UV light.

The optical constants (refractive index, *n*, and extinction coefficient *k*) of the pure host materials, TPBi and PFO (Fig. S1 and S2, ESI†), were determined experimentally by spectroscopic ellipsometry at various angles of incidence and transmission intensity measurements at 0° (perpendicular incidence). Optical constants for the host material containing also molecular emitter (<10 wt%) were assumed to be the same as the pure host. Optical constants were determined using WVASE 32 software from Woollam. Ellipsometry on layers of the pure TPBi did not reveal any indication for optical anisotropy, therefore the optical properties of the host were taken to be the same in all directions. For PFO, uniaxial optical anisotropy was taken in consideration and ordinary and extraordinary optical constants were extracted from ellipsometry data of the pure host polymer.

The experimental angular dependencies of the *p*-polarised fluorescence intensities were used as input for optical simulations using the software OrientExpress. The angular emission profile of the vertical and horizontal radiating dipoles is simulated based on the thickness and (anisotropic) refractive indices of all layers and the substrate which define a microcavity. Hereby an even distribution of the emitters across the emitting layer is assumed and the intensities of the contributing dipoles are added accordingly. The relative weights of the contributions of horizontal and vertical dipoles to the emission profile are then obtained from the measured one by simple linear regression. The details can be found in ESI,† Note 1. The comparison of the experimental data with the optical simulations allows one to quantify the degree of orientation of the transition dipole moments of the emitters in the host.

## Results and discussion

### Fluorescence of PFO:guest blends

**Fig. 3:**
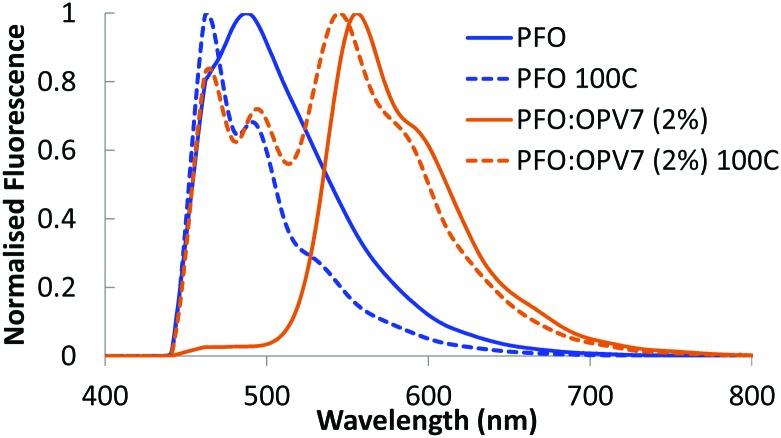
Fluorescence of PFO (in blue) and PFO:OPV7 (2%) (in orange) for layers which are not annealed (solid lines) and layers annealed at 100 °C (dashed lines). The measurements were taken after samples had cooled down to room temperature, with the angle dependent fluorescence measurement setup at 0°.

Upon admixing a small amount (2 wt%) of OPV7 oligomer into the PFO host matrix, the bright yellow-orange fluorescence of the oligomer becomes apparent. In Fig. 3, we show the normalized fluorescence spectrum of the as cast PFO:OPV7 blend with a maximum at 560 nm. The pure PFO host also shows fluorescence with the main emission band in the UV spectral range around 410 nm, just outside the window of observation. The long wavelength tail of the PFO emission is however clearly visible in the spectrum of the pure polymer (Fig. 3). In the not-annealed PFO:OPV7 blend, the luminescence from the host polymer is strongly suppressed, most likely by energy transfer from the host to the guest.

**Fig. 4:**
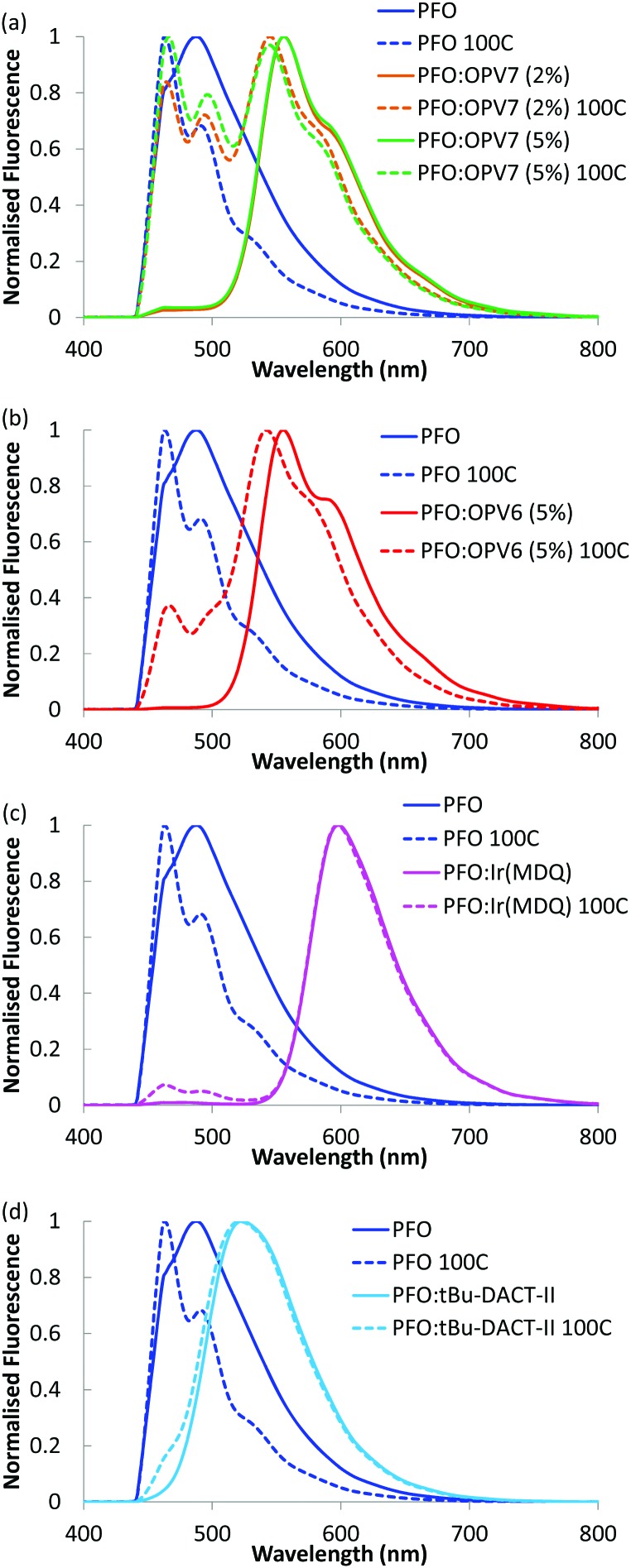
Fluorescence of different blends in PFO. Fluorescence of PFO in blue; non annealed layers (solid lines) and layers annealed at 100 °C (dashed lines). PFO:OPV7 (2%) in orange (a); PFO:OPV7 (5%) in green (a); PFO:OPV6 (5%) in red (b); PFO:Ir(MDQ)_2_(acac) in pink (c); PFO:*t*Bu-DACT-II in light blue (d). The measurements were taken after cooling of the samples to room temperature, with the angle dependent fluorescence measurement setup at 0°.

In order to enhance the alignment of polymer and guest, the films were thermally annealed. Thermal annealing has a strong influence of the fluorescence characteristics of the pure PFO film.^32^ The emission maximum shifts to longer wavelength and now appears around 440 nm.^33^ Changes in the fluorescence spectrum of PFO have been studied in detail and the shift of the fluorescence maximum has been attributed to formation of domains of β-phase of the polymer within the layer.^34^ For the thermally annealed PFO:OPV7 film (Fig. 3), the emission of the OPV7 is slightly blue shifted compared to the as-cast film. This blue shift, which may be opposite to what is expected for annealed films, can be attributed to two main factors. After annealing, the emission from PFO overlaps with the peak emission wavelength for OPV7, causing a small shift. Second, annealing causes a change in the polarizability of the host, and this can influence the energy of the excited states, therefore the emission wavelength.^35^ We note that this shift also signifies that aggregation of OPV7 in PFO does not occur by the thermal annealing, because that would lead to a red shift of the fluorescence.^20^ The intensity of the luminescence from PFO relative to the OPV7 signal has increased upon thermal annealing (Fig. 3). This may be interpreted in terms of an increase in the probability for photon emission by the pure host and/or phase segregation of nanocrystalline PFO domains in the film. Atomic force microscopy images of the PFO:OPV7 films of both the annealed and non-annealed layers show a flat topography with a low root mean squared surface roughness *R*_q_ of <1 nm (ESI,† Fig. S3). Finally, we observed that upon increasing the loading of the PFO host with OPV7 guest to 5 wt%, the fluorescence bands of the OPV7 do not change appreciably in either position or in intensity relative to the PFO (see Fig. 4a). The insensitivity of the OPV7 fluorescence bands to changes in the loading, strongly indicates that a possible contribution from aggregates of the OPV7 to the total emission spectrum is insignificant. The photoluminescence spectra of the PFO:OPV6, PFO:Ir(MDQ)_2_(acac) and PFO:*t*Bu-DACT-II blends are shown in Fig. 4. Also for these systems the emission from the guest can be clearly distinguished from the fluorescence from the host. The band-shape and spectral position of the luminescence from the guests do not change appreciably upon annealing of the mixed systems, which is consistent with absence of aggregation of the guest upon thermal treatment.

### Angular dependent fluorescence

**Fig. 5:**
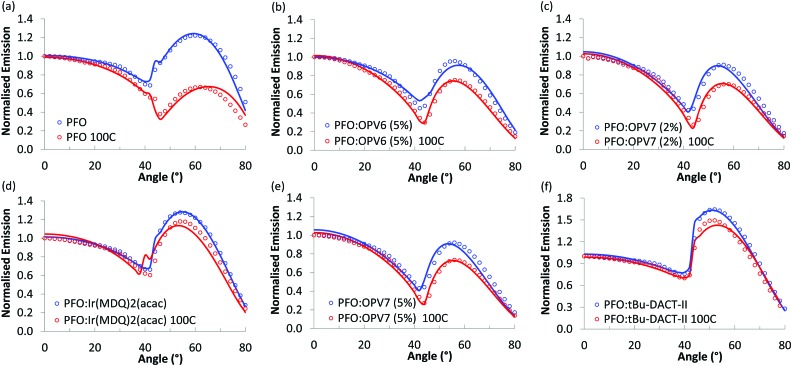
Angular dependence of the *p*-polarised fluorescence of solution processed layers of host/guest systems with PFO as host material, normalised at 0°, for both non-annealed layers (blue) and layers annealed at 100 °C (red). The dots represent the experimental measurements, the full lines the simulated results. In all the cases, the annealed films, compared with the not annealed ones, show a lower peak at high angles (52° to 60°), meaning a more horizontal orientation of the emitters in the annealed films. The difference between annealed and not annealed films is higher for pristine PFO and for the two oligomers, but smaller for the smaller molecules.

Fig. 5 shows the angular dependence of the *p*-polarised fluorescence of the pure PFO host and of the host/guest systems. Results for both as cast films (blue markers) and thermally treated layers (red markers) are given. For each film, the angular dependence of the luminescence was probed at the wavelength of maximum intensity and normalized to the intensity of the luminescence in the direction of the surface normal of the film (0°). For all the films, the fluorescence intensity slowly decreases when the angle is varied from 0° up to ∼42°, and then rapidly increases, with a broad maximum at angles between 52° and 60°, depending on the host/guest system under study. The minimum in *p*-polarised fluorescence intensity at 42° is related to the Brewster angle for internal reflection at the host/air interface where light can couple out effectively in the backward direction.

We first focus on the results for the pure PFO layer. As can be seen, the angular distribution of the fluorescence is strongly influenced by thermal annealing of the polymer. While the as-cast film shows a maximum at 60°, for the annealed layer the intensity at high angles is much lower than that for directions near the normal. To rationalize these results, we note that a transition dipole moment oriented along the normal of the film would radiate waves with propagation direction predominantly in the plane of the film. The vertical orientation of emitter dipoles thus should result in high intensities for large angles in the angular emission profile. In contrast, a transition dipole moment oriented in plane with the film, would emit in the direction normal to the plane, and would thus show low intensity at high angles in the angle dependent measurements. Thus, the low intensity observed for the thermally annealed PFO at ∼60° is consistent with highly horizontally aligned emitters in the film. We note that for PFO due to the presence of domains of β-phase the orientational distribution of fluorescent sites may not be representative for the complete set of polymer chains.

In order to quantify the orientation of the transition dipole moments, we combine the measurements with optical simulation of the angular intensity profile of the *p*-polarized emission. With the software OrientExpress, the simulated angular emission profile of the vertical and horizontal radiating dipoles is fitted to the measured ones by weighting their respective contributions (more details in ESI,† Note 1). For a fully random distribution of transition dipole moments, the coefficients for vertical and horizontal orientation are *θ*_v_ = 0.33 and *θ*_h_ = 0.66, respectively.

The simulated results show an excellent fit with the experimental data (Fig. 5) and the simulated values for vertical orientation of transition dipole moments in the analysed layers are summarised in Table 1. The non-annealed PFO layer shows a slightly horizontal orientation of the average transition dipole moment, that becomes almost completely horizontal (*θ*_v_ = 0.06) after annealing of the layer.

**Table 1:** Coefficient for vertical orientation (*θ*_v_) for transition dipole moments in PFO and PFO/guest layers^*a*^

Material	*θ*_v_ not annealed	*θ*_v_ annealed
PFO	0.26	0.06
PFO:OPV7 (2%)	0.19	0.10
PFO:OPV7 (5%)	0.19	0.12
PFO:OPV6 (5%)	0.17	0.14
PFO:Ir(MDQ)_2_(acac) (10%)	0.29	0.22
PFO:*t*Bu-DACT-II (9%)	0.42	0.43

^*a*^Obtained by fitting the experimental angular *p*-polarised fluorescence intensity to simulated profiles for partial horizontal (*θ*_h_) and vertical (*θ*_v_) orientation. The values are for both not annealed and annealed layers.

The blends of PFO with the two oligomers OPV6 and OPV7 already show appreciable horizontal orientation in the as-cast state. The horizontal orientation improves upon thermal annealing, but the effect of the annealing is less strong than for pure PFO. Ir(MDQ)_2_(acac) is almost randomly oriented in the as-cast film, but its transition dipoles become more parallel to the plane of the film upon thermal treatment (*θ*_v_ = 0.22). The degree of horizontal orientation in these solution processed PFO:Ir(MDQ)_2_(acac) films is very similar to that of Ir(MDQ)_2_(acac) in co-evaporated layers with TPBi where *θ*_v_ = 0.23 (Fig. S4 and Table S1, ESI†). Finally, the *t*Bu-DACT-II molecules show a preference for vertical orientation of their transition dipole in both as cast and annealed films. This is the opposite result to DACT-II thin-films fabricated by vacuum-deposition method, which prefer horizontal orientation with *θ*_v_ = 0.26.^8^

**Fig. 6:**
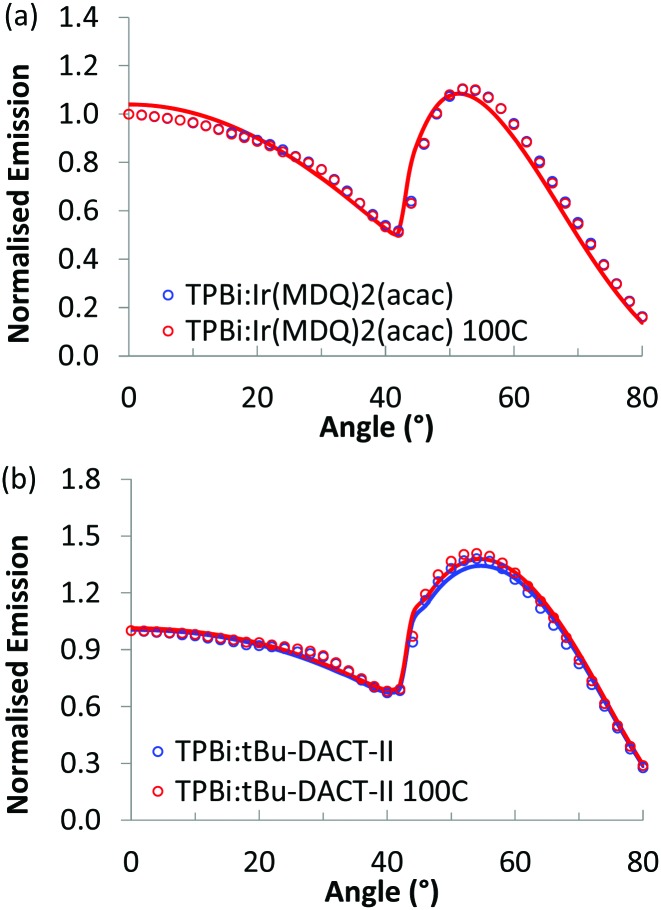
Angular dependence of the *p*-polarised fluorescence of solution processed layers of host/guest systems with TPBi as host material, normalised at 0°, for both not annealed layers (blue) and layers annealed at 100 °C (red). The dots represent the experimental measurements, the full lines the simulated results. There is no difference between annealed and non-annealed films.

As a control experiment, we also investigated the emitter dipole orientation of Ir(MDQ)_2_(acac) and *t*Bu-DACT-II in TPBi, a host matrix that shows no appreciable orientation. Results are shown in Fig. 6 and summarized in Table 2. For Ir(MDQ)_2_(acac) the orientation coefficient of the transition dipole moments is consistent with fully random orientation. This holds both for as-cast and thermally annealed films. For *t*Bu-DACT-II in TPBi, the data indicate a small preference for vertical orientation, similar to in the polymer host (Table 1). In a previous study, the orientation of OPV6 and OPV7 in TPBi was already investigated. Under conditions where aggregation of the OPV6 and OPV7 in the TPBi host are minimized, the oligomers orient randomly in the solution processed small molecules host (*θ*_v_ = 0.32 and *θ*_v_ = 0.34).^20^

**Table 2:** Coefficient for vertical orientation (*θ*_v_) for transition dipole moments in TPBi:Ir(MDQ)_2_(acac) and TPBi:*t*Bu-DACT-II solution processed thin films

Material	*θ*_v_ non-annealed	*θ*_v_ annealed
TPBi:Ir(MDQ)_2_(acac) (10%)	0.34	0.34
TPBi:*t*Bu-DACT-II (9%)	0.41	0.42

## Discussion

**Fig. 7:**
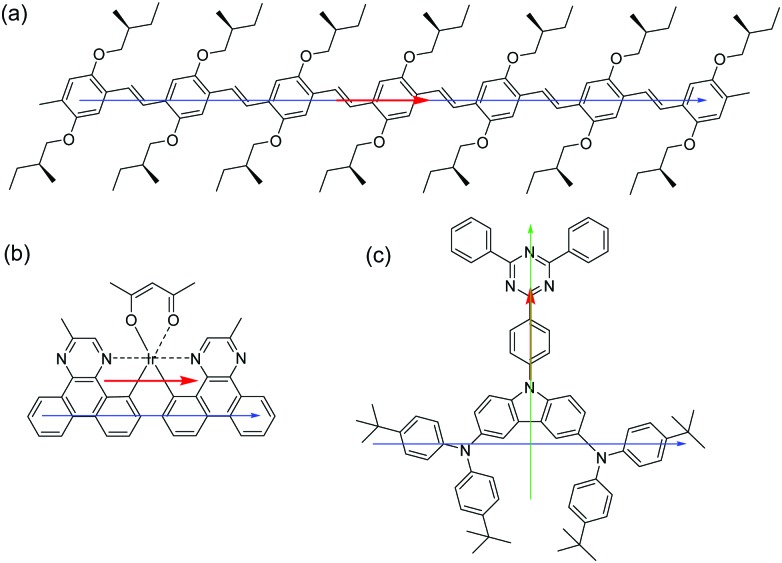
Chemical structures of the emitters used in this work (a) OPV7, (b) Ir(MDQ)2(acac), (c) *t*Bu-DACT-II with approximations of their longest backbone axis (blue and green) and the direction of transition dipole moment (red).

The results demonstrate that the emitters with the most elongated backbone (OPV6 and OPV7) also show the highest degree of horizontal orientation of the transition dipole moment. In OPV molecules the transition dipole moment is mainly oriented along the long axis of the molecules (Fig. 7a).^24^ The combination of a high structural aspect ratio and a transition dipole moment along the long axis are a winning combination.

For heteroleptic iridium based emitters two factors were previously recognised to be important for horizontal orientation of the average transition dipole moment when deposited *via* thermal evaporation.^28^ First, the emitter should have a triplet transition dipole moment preferentially oriented along a specific direction and not have a combination of transition dipole moments with various orientations, and second, the emissive molecule itself should have a preferred orientation with respect to the substrate, in such a way that the combination of both conditions leads to transition dipole moments that are parallel to the horizontal direction. For the *N*,*N-trans* isomer of Ir(MDQ)_2_(acac) the average transition dipole moment from the luminescent transition for the excited triplet level was calculated to be oriented approximately parallel to the longest axis of the molecule (Fig. 7b).^28^ The preferential orientation in the annealed PFO layer may thus be rationalized in terms of the main transition being parallel to the longest axis of the molecule and the longest axis of the molecule aligning with the PFO chains.

*t*Bu-DACT-II does not have an elongated shape, and it is difficult to identify the longest axis in the molecule. It may be along the donor–acceptor direction (green arrow in Fig. 7c) but could also be along a line joining the two triarylamine moieties (blue arrow in Fig. 7c). The direction of the transition dipole moment for the S_1_–S_0_ transition is most likely from the carbazole to the triazine ring. Our measurements of the emission anisotropy indicate that the *t*Bu-DACT-II molecules orient with their transition dipole moment (*i.e.* the carbazole–triazine direction) perpendicular to the direction of the PFO chains. If we consider the long axis of the molecule as the blue arrow in Fig. 7, this is in line with what observed for the other emitters.

Comparison of the orientation of OPV6 and OPV7 in an isotropic small molecular host as TPBi thus supports the view that the orientational order of the polymeric host is instrumental in aligning the emitter molecules predominantly in the horizontal plane. The experimental result that for the small molecular emitters Ir(MDQ)_2_(acac) and *t*Bu-DACT-II the nature of the host has a much smaller influence on the orientation of the guest indicates that the aspect ratio of the guest is an important factor. The larger the aspect ratio of the guest, the more susceptible it seems to be for directional influence of the polymer host on its orientation. Hence, the high preference for horizontal orientation of OPV6 or OPV7 correlates with the high aspect ratio of these molecules. For evaporated layers a correlation between aspect ratio of the molecules and preferential horizontal orientation has also been proposed.^6^ We note however that the underlying physical mechanism for inducing the order may be different for solution and gas phase processing.^15^

## Conclusion

In solution processed layers of host/guest systems, relevant for OLED applications, the orientation of small molecular emitters can be influenced by using a polymeric host that shows preferential alignment in the horizontal plane. The experimental evidence obtained suggests that rod like emitter molecules adopt a preferential horizontal orientation under the influence of the host. In particular, for the oligomers OPV6 and OPV7 in a PFO host, it was possible to reach extremely high horizontal orientation (with 86 to 90% of dipoles horizontally oriented) after thermal annealing, without detrimental aggregation of the luminescent guest molecules. For solution processed layers of Ir(MDQ)_2_(acac) in PFO, after thermal annealing, it was possible to reach the same degree of orientation observed for the same emitter in evaporated layers. The use of poly(9,9-dioctylfluorene) as host material in host/guest systems seems to be a good way to achieve horizontal orientation of transition dipole moments in solution processed oligomers and small molecular emitters. The generalization of this result to other polymeric hosts is under investigation.

## Supplementary Material

Supplementary information

## References

[cit1] Kato K., Iwasaki T., Tsujimura T. (2015). J. Photopolym. Sci. Technol..

[cit2] Meerheim R., Furno M., Hofmann S., Lüssem B., Leo K. (2010). Appl. Phys. Lett..

[cit3] Liehm P., Murawski C., Furno M., Lüssem B., Leo K., Gather M. C. (2012). Appl. Phys. Lett..

[cit4] Frischeisen J., Yokoyama D., Endo A., Adachi C., Brütting W. (2011). Org. Electron..

[cit5] Flämmich M., Frischeisen J., Setz D. S., Michaelis D., Krummacher B. C., Schmidt T. D., Brütting W., Danz N. (2011). Org. Electron..

[cit6] Yokoyama D. (2011). J. Mater. Chem..

[cit7] Kim S.-Y., Jeong W.-I., Mayr C., Park Y.-S., Kim K.-H., Lee J.-H., Moon C.-K., Brütting W., Kim J.-J. (2013). Adv. Funct. Mater..

[cit8] Kaji H., Suzuki H., Fukushima T., Shizu K., Suzuki K., Kubo S., Komino T., Oiwa H., Suzuki F., Wakamiya A., Murata Y., Adachi C. (2015). Nat. Commun..

[cit9] Kuei C.-Y., Tsai W.-L., Tong B., Jiao M., Lee W.-K., Chi Y., Wu C.-C., Liu S.-H., Lee G.-H., Chou P.-T. (2016). Adv. Mater..

[cit10] Lin T.-A., Chatterjee T., Tsai W.-L., Lee W.-K., Wu M.-J., Jiao M., Pan K.-C., Yi C.-L., Chung C.-L., Wong K.-T., Wu C.-C. (2016). Adv. Mater..

[cit11] Kim K.-H., Moon C.-K., Lee J.-H., Kim S.-Y., Kim J.-J. (2014). Adv. Mater..

[cit12] Komino T., Sagara Y., Tanaka H., Oki Y., Nakamura N., Fujimoto H., Adachi C. (2016). Appl. Phys. Lett..

[cit13] Mayr C., Lee S. Y., Schmidt T. D., Yasuda T., Adachi C., Brütting W. (2014). Adv. Funct. Mater..

[cit14] Lampe T., Schmidt T. D., Jurow M. J., Djurovich P. I., Thompson M. E., Brütting W. (2016). Chem. Mater..

[cit15] Jurow M. J., Mayr. C., Schmidt T. D., Lampe T., Djurovich P. I., Brütting W., Thompson M. E. (2016). Nat. Mater..

[cit16] McBranch D., Campbell I. H., Smith D. L., Ferraris J. P. (1995). Appl. Phys. Lett..

[cit17] Sturm J., Tasch S., Niko A., Leising G., Toussaere E., Zyss J., Kowalczyk T. C., Singer K. D., Scherf U., Huber J. (1997). Thin Solid Films.

[cit18] Tammer M., Monkman A. P. (2012). Adv. Mater..

[cit19] Ramsdale C. M., Greenham N. C. (2012). Adv. Mater..

[cit20] Senes A., Meskers S. C. J., Dijkstra W. M., van Franeker J. J., Altazin S., Wilson J. S., Janssen R. A. J. (2016). J. Mater. Chem. C.

[cit21] Zhao L., Komino T., Inoue M., Kim J.-H., Ribierre J. C., Adachi C. (2015). Appl. Phys. Lett..

[cit22] Zhao L., Komino T., Kim D. H., Sazzad M. H., Pitrat D., Mulatier J.-C., Andraud C., Ribierre J.-C., Adachi C. (2016). J. Mater. Chem. C.

[cit23] Whitehead K. S., Grell M., Bradley D. D. C. (2000). Appl. Phys. Lett..

[cit24] Chandross M., Mazumdar S., Liess M., Lane P. A., Vardeny Z. V., Hamaguchi M., Yoshino K. (1997). Phys. Rev. B: Condens. Matter Mater. Phys..

[cit25] Penninck L., Steinbacher F., Krause R., Neyts K. (2012). Org. Electron..

[cit26] Schmidt T. D., Setz D. S., Flämmich M., Frischeisen J., Michaelis D., Krummacher B. C., Danz N., Brütting W. (2011). Appl. Phys. Lett..

[cit27] Graf A., Liehm P., Murawski C., Hofmann S., Leo K., Gather M. C. (2014). J. Mater. Chem. C.

[cit28] Kim K.-H., Lee S., Moon C.-K., Kim S.-Y., Park Y.-S., Lee J.-H., Lee J. W., Huh J., You Y., Kim J.-J. (2014). Nat. Commun..

[cit29] Goushi K., Yoshida K., Sato K., Adachi C. (2012). Nat. Photonics.

[cit30] Peeters E., Janssen R. A. J., Meskers S. C. J., Meijer E. W. (1999). Polym. Prepr..

[cit31] Frischeisen J., Yokoyama D., Adachi C., Brütting W. (2010). Appl. Phys. Lett..

[cit32] Campoy-Quiles M., Etchegoin P. G., Bradley D. D. C. (2005). Synth. Met..

[cit33] Grell M., Bradley D. D. C., Ungar G., Hill J., Whitehead K. S. (1999). Macromolecules.

[cit34] Grell M., Bradley D. D. C., Inbasekaran M., Ungar G., Whitehead K. S., Woo E. P. (2000). Synth. Met..

[cit35] Suppan P. (1990). J. Photochem. Photobiol., A.

