# Supplementary material for: Increasing the horizontal orientation of transition dipole moments in solution processed small molecular emitters
Source: J Mater Chem C Mater. 2017 Jun 12;5(26):6555–62. doi: 10.1039/c7tc01568b (PMC5735365; doi:10.1039/c7tc01568b)
Supplement: Supplementary file 1 [file TC-005-C7TC01568B-s001.pdf]

Electronic Supplementary Material (ESI) for Journal of Materials Chemistry C.
This journal is © The Royal Society of Chemistry 2017

Electronic Supplementary Information

Increasing the horizontal orientation of transition dipole moments in solution processed small molecular emitters

Alessia Senes, Stefan C. J. Meskers, Horst Greiner, Katsuaki Suzuki, Hironori Kaji, Chihaya Adachi, Joanne S. Wilson and René. A. J. Janssen

Synthesis of tBu-DACT-II

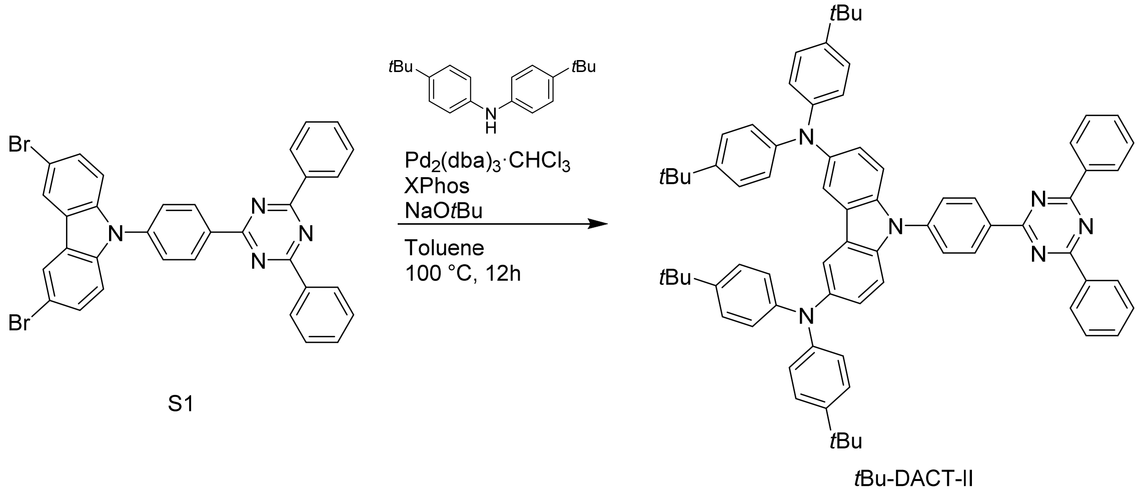

Scheme S1. Synthesis of tBu-DACT-II.

S1 (300 mg, 0.48 mmol), $Pd_2(dba)_3\cdot CHCl_3$ (12.4 mg, 12.0 µmol), XPhos (22.9 mg, 48.0 µmol), NaO*t*-Bu (110 mg, 1.15 mmol), and bis(4-(tert-butyl)phenyl)amine (298 mg, 1.06 mmol) was dissolved in dry and deoxygenated toluene (1 mL). The mixture was stirred at 100 °C for 12 h and allowed to cool to room temperature. The reaction mixture was poured into water and extracted with CH_2Cl_2 (10 mL × 3). The organic phase was dried over Na_2SO_4, filtered off, and concentrated under reduced pressure. The resulting crude product was purified by silica gel column chromatography (CH_2Cl_2 : hexane = 1 : 5) to give 441 mg (0.43 mmol) of ***t*Bu-DACT-II** in 89% yield as yellow solids.

^{1}H NMR (800 MHz, $CDCl_3$): δ 9.00 (d, J = 8.5 Hz, 2H), 8.81 (d, J = 7.0 Hz, 4H), 7.82 (d, J = 8.5 Hz, 2H), 7.81 (d, J = 2.2 Hz, 16.8 Hz, 2H), 7.22 (d, J = 8.7 Hz 8H), 7.01 (d, J = 8.7 Hz, 8H), 1.30 (s, 36H); ^{13}C NMR (201 MHz, $CDCl_3$): δ 171.8, 170.9, 145.9, 144.3, 141.7, 141.3, 137.7, 136.2, 134.7, 132.7, 130.6, 129.0, 128.7, 126.4, 125.9, 125.8, 124.6, 122.2, 118.3, 110.9, 34.18, 34.46; APCI-MS (m/z): $[M+H]^+$ calcd. for $C_{43}H_{34}N_3$, 1033.5897; found, 1033.5849.

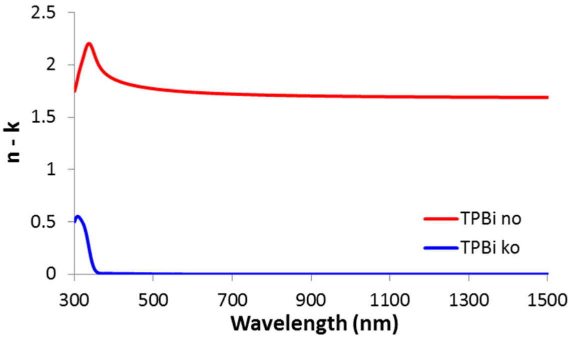

Fig. S1 Refractive index and extinction coefficient of TPBi

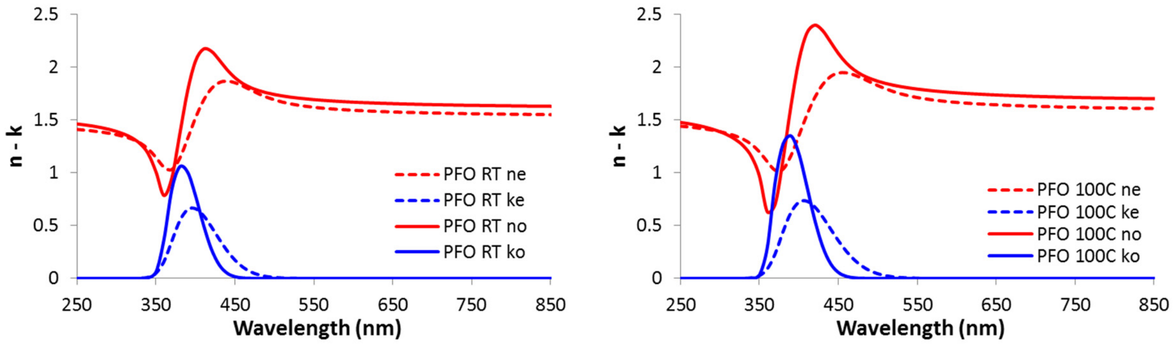

Fig. S2 Ordinary and extraordinary refractive index and extinction coefficient of PFO, not annealed (left) and annealed (right)

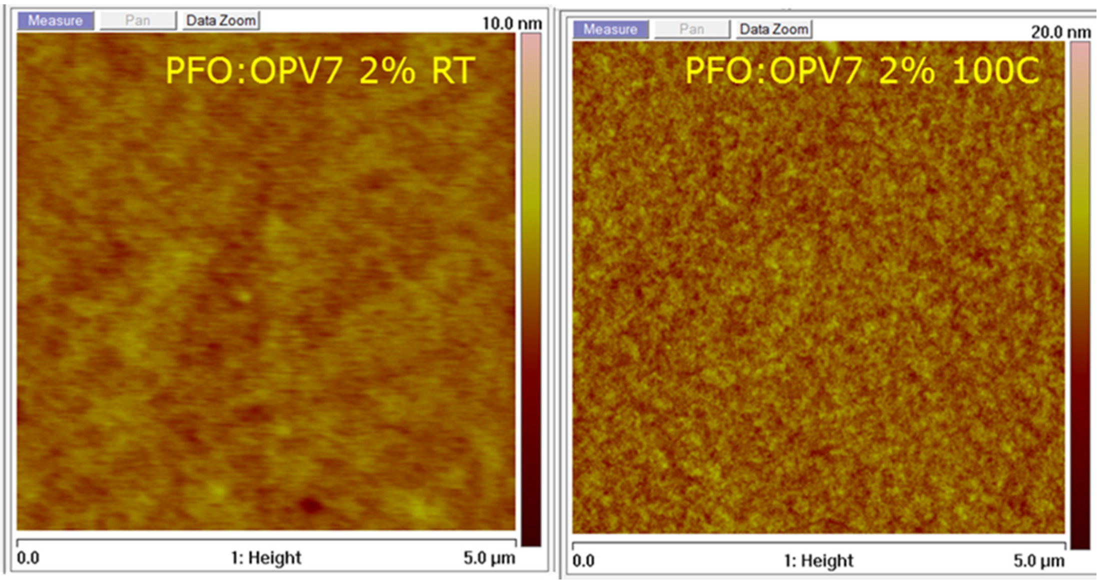

Fig. S3 AFM topography scans (height images) for 30 nm thick films of PFO:OPV7 (2%): layer not annealed (left) and annealed at 100 °C (right)

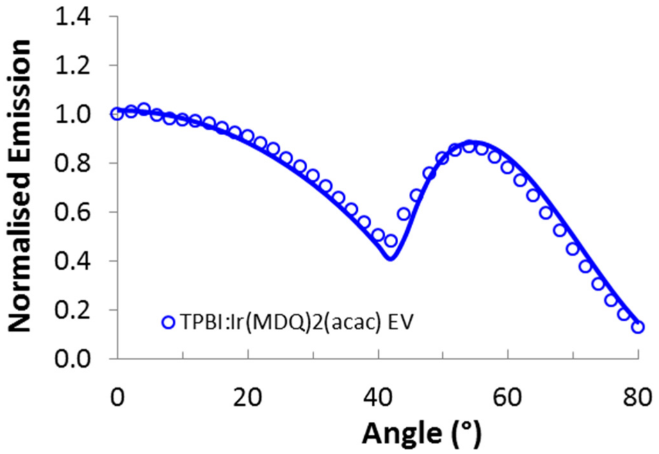

Fig. S4 Angular dependence of the p-polarised photoluminescence of an evaporated TPBi:Ir(MDQ)$_2$(acac) 40 nm thick layer. The dots represent the experimental measurements, the full lines the simulated results.

Table S1 Coefficient for vertical orientation (θ_v) of transition dipole of an evaporated TPBi:Ir(MDQ)$_2$(acac) 40 nm thick layer. The sample was not annealed (annealing did not cause any change)

Material	θ_v Non-annealed
TPBi:Ir(MDQ)$_2$(acac) Evaporated	0.23

Note1: Description of OrientExpress (Horst Greiner, horst.greiner@netaachen.de)

To determine the orientation parameter of an ensemble of radiating dipoles the simulated angular emission profile of the vertical and horizontal has to be fitted to the measured one by weighing their respective contributions. Therefore it is primordial to calculate the emission profiles in a correct way. For an isotropic emission medium the appropriate formulas are given in Neyts [S1]. Moon [S2], Penninck [S3] and Wasey [S4] treat the anisotropic case, but do not provide explicit formulas. Here we derive and display the correct expressions by applying an elegant "scaling" procedure due to Clemmow [S5] starting from the electrical fields of a dipole in vacuum. Finally it is shown how the orientation parameter can be obtained from simple linear regression.

We first recapitulate the isotropic case and then show how it can be generalized to the anisotropic one. The presentation follows the book by Novotny and Hecht [S6].

The power and its angular distribution P(**p**) emitted by a dipole **p**=|p|**n** with a direction unit vector **n** located at the coordinate origin in an infinite isotropic medium of refractive index n_o is given by the formula

(1) $$P(\mathbf{p}) = \frac{\omega}{2}\mathrm{Im}(\overline{\mathbf{p}} \bullet \mathbf{E}(0))$$

where $\mathbf{E}$ is the electrical field generated by the dipole evaluated at the coordinate origin and ω its oscillation frequency, see [S6], chapter 8.3. $\overline{\mathbf{p}}$ denotes the complex conjugate of $\mathbf{p}$. The electrical field at a location (*x*, *y*, *z*) can be represented by the angular spectrum representation of the Greens tensor of the infinite medium and after some juggling with constants can be written as

(2) $$\mathbf{E}(x,y,z) = \frac{\omega^3 |p|}{6\pi\ c^3 \varepsilon_0} \frac{3}{4\pi} \mathrm{Im}(i\int_{-\infty}^{-\infty}\int_{\infty}^{\infty} \mathbf{M}(k_x,k_y)\mathbf{n}\ e^{k_x x + k_y y + k_z z} dk_x dk_y)$$
$$\text{with } k_z = \sqrt{n_o^2 - (k_x^2 + k_y^2)}$$

where the constants have been arranged for later convenience. $\mathbf{M}$ is a 3 by 3 matrix whose entries depend on k_x and k_y. In the sequel all wave vectors are dimensionless and normalized to the wave vector in vacuum $k_{vac} = \frac{\omega}{c}$.

As demonstrated in [S5, S6] the electrical field can be decomposed in a TE and TM part in a standard way and the following expressions can be derived:

As the TE component of the electrical field does not involve the extraordinary index we obtain

(3) $$\mathbf{E}^{TE}(x,y,z) = \frac{\omega^3 |p|}{6\pi\ c^3 \varepsilon_0} \frac{3}{4\pi} \mathrm{Im}(i\int_{-\infty}^{-\infty}\int_{\infty}^{\infty} \mathbf{M}^{TE}(k_x,k_y)\mathbf{n}\ e^{k_x x + k_y y + k_z z} dk_x dk_y)$$

where

(4) $$\mathbf{M}^{TE}(k_x,k_y) = \frac{1}{k_z(k_x^2 + k_y^2)} \begin{bmatrix} k_y^2 & -k_x k_y & 0 \\ -k_x k_y & k_x^2 & 0 \\ 0 & 0 & 0 \end{bmatrix} \quad \text{and } k_z = \sqrt{n_0^2 - (k_x^2 + k_y^2)}, \quad \mathrm{Im}(k_z) \geq 0$$

which is identical to the formula given by [S6], chapter 10.3 for the isotropic case.

To derive the TM part of the field which involves the ordinary and extraordinary index we employ the scaling of the spatial coordinates (x, y, z) of the electrical field and the dipole sources given by [S5] to the TM field in vacuum ($n_o = n_e = 1$) as given in [S6], chapter 10.2.

Introducing these expressions in the angular spectrum representation (5) and performing the change of variables $k_x \to k_x / n_e \quad k_y \to k_y / n_e$ in the integrand the angular spectrum representation of the TM part of the field transforms to

$$(5) \quad \mathbf{E}^{TM}(x,y,z) = \frac{\omega^3 |p|}{6\pi\, c^3 \varepsilon_0} \frac{3}{4\pi} \mathrm{Im}(i \int_{-\infty}^{-\infty} \int_{\infty}^{\infty} \mathbf{M}^{TM}(k_x,k_y)\mathbf{n}\, e^{k_x x + k_y y + k_z z} dk_x dk_y)$$

where

$$(6) \quad \mathbf{M}^{TM}(k_x,k_y) = \frac{1}{(k_x^2+k_y^2)} \begin{bmatrix} \frac{k_x^2 k_z}{n_o^2} & \frac{k_x k_y k_z}{n_o^2} & \frac{\mp k_x (k_x^2+k_y^2)}{n_e^2} \\ \frac{k_x k_y k_z}{n_o^2} & \frac{k_y^2 k_z}{n_o^2} & \frac{\mp k_y (k_x^2+k_y^2)}{n_e^2} \\ \mp\frac{1}{n_e^2} k_x (k_x^2+k_y^2) & \mp\frac{1}{n_e^2} k_y (k_x^2+k_y^2) & \frac{n_0^2}{n_e^4} \frac{(k_x^2+k_y^2)^2}{k_z} \end{bmatrix}$$

$$\text{and } k_z = \sqrt{n_0^2 - \frac{n_0^2}{n_e^2}(k_x^2+k_y^2)}, \quad \mathrm{Im}(k_z) \geq 0 \quad \text{and } z \geq 0:\ - \quad z \leq 0:\ +$$

With these expressions the angular power spectrum of the vertical and horizontal dipoles can easily be calculated by inserting the angular spectrum for *x* = *y* = *z* = 0 into (1) and evaluating the resulting integral. In the sequel we abbreviate

$$P_{vac} = \frac{\omega^4 |p|^2}{12\pi\, c^3 \varepsilon_0}$$

for the power radiated by a dipole in vacuum.

For the TE part of a horizontal dipole pointing in the x direction we calculate

$$(7) \quad P_{hor}^{TE} = P_{vac} \frac{3}{4\pi} \mathrm{Im}(i \int_{-\infty}^{-\infty} \int_{\infty}^{\infty} \frac{k_y^2}{k_z (k_x^2+k_y^2)} dk_x dk_y) = P_{vac} \frac{3}{4} \int_0^{n_o} \frac{\kappa}{\sqrt{n_o^2 - \kappa^2}} d\kappa = \frac{3}{4} P_{vac} n_o$$

where the substitution $k_x = \kappa \cos\psi \quad k_y = \kappa \sin\psi$ has been applied to evaluate the integral.

The integral extends only to n_o as for $\kappa > n_o$ the integrand becomes imaginary.

For the TM part of a vertical dipole we get in a similar way

$$(8)\quad P_{ver}^{TM} = P_{vac}\,\frac{3}{4\pi}\,\mathrm{Im}(i\int_{-\infty}^{-\infty}\int_{\infty}^{\infty}\frac{n_0^2}{n_e^4}\frac{(k_x^2+k_y^2)}{k_z}\,dk_x dk_y) = P_{vac}\,\frac{3}{2}\frac{n_0}{n_e^3}\int_0^{n_e}\frac{\kappa^3}{\sqrt{n_e^2-\kappa^2}}d\kappa = P_{vac}n_o$$

We see that the total radiated power is equal to the power radiated in an isotropic medium.

For the TM part of a horizontal dipole say along the *x*-axis we obtain

$$(9)\quad P_{hor}^{TM} = P_{vac}\,\frac{3}{4\pi}\,\mathrm{Im}(i\int_{-\infty}^{-\infty}\int_{\infty}^{\infty}\frac{k_x^2 k_z}{n_o^2(k_x^2+k_y^2)}dk_x dk_y) = P_{vac}\,\frac{3}{4}\frac{1}{n_o^2}\frac{n_o}{n_e}\int_0^{n_e}\kappa\sqrt{n_e^2-\kappa^2}\,d\kappa = \frac{1}{4}P_{vac}\frac{n_e^2}{n_o}$$

Apparently the total radiated power differs from the isotropic case where it amounts to $\frac{1}{4}P_{vac}n_o$.

The main result of our analysis is that the "TM power densities" $P^{TM}(\kappa)$ for the horizontal and vertical dipoles for the anisotropic case can be obtained from the ones applicable to the isotropic case by replacing $k_z = \sqrt{n_o^2 - \kappa^2}$ with $k_z = \sqrt{n_o^2 - \frac{n_o^2}{n_e^2}\kappa^2}$ and by multiplication with $(\frac{n_o}{n_e})^4$ in the case of vertical dipoles.

With these expressions for the dipole powers as a function of κ the intensities emitted into the quartz substrate can easily obtained, see e.g. [S1] as the emitter material and the quartz and air substrate form a micro cavity. To calculate the power emitted into the quartz substrate the dipole power has to be multiplied by a micro cavity factor which depends on κ and involves interference factors of the form

$$(10)\quad a_{subs}(\kappa) = r_{subs}(\kappa)\exp(4\pi i k_{z,EML} z_{dipole}/\lambda)$$

for the EML substrate interface where in our case subs stands for the quartz or air substrate. z_{dipole} denotes the distance of the emitting dipole to the EML/substrate interface and

$$(11)\quad k_{z,EML}^{TE} = \sqrt{n_o^2-\kappa^2} \qquad k_{z,EML}^{TM} = \sqrt{n_o^2 - \frac{n_o^2}{n_e^2}\kappa^2} \qquad k_{z,subs} = \sqrt{n_{subs}^2-\kappa^2}$$

The first factor represents the TE or TM reflectivity at the EML/substrate interface.

$$(12)\quad r_{subs}^{TE}(\kappa) = \frac{k_{z,EML}^{TE} - k_{z,subs}^{TE}}{k_{z,EML}^{TE} + k_{z,subs}^{TE}} \qquad r_{subs}^{TM}(\kappa) = \frac{n_{subs}^2 k_{z,EML}^{TM} - n_o^2 k_{z,subs}^{TM}}{n_{subs}^2 k_{z,EML}^{TM} + n_o^2 k_{z,subs}^{TM}}$$

In this formula the ordinary index n_o has to be used as the refractive index of the anisotropic layer, see Yeh, chapter 9.6, [S7].

The TM part of the radiant intensity of the vertical dipoles in the quartz substrate for an emission angle θ is then given by (setting $\kappa = n_{quartz}\sin\theta$ and using $d\kappa = n_{quartz}\cos\theta$), see [S1]:

(13) $$I_{ver}^{TM}(\theta)2\pi\sin\theta d\theta = P_{vac}(1-r_{quartz}^2(\kappa))\left|\frac{1+a_{air}(\kappa)}{1-a_{air}(\kappa)a_{quartz}(\kappa)}\right|^2 \frac{3}{4\pi}\frac{n_0^2 n_{quartz}^2\cos\theta}{n_e^4}\frac{\kappa^2}{\sqrt{n_o^2-\frac{n_o^2}{n_e^2}\kappa^2}}2\pi\sin\theta d\theta$$

and similarly for horizontal ones by

(14) $$I_{hor}^{TM}(\theta)2\pi\sin\theta d\theta = P_{vac}(1-r_{quartz}^2(\kappa))\left|\frac{1-a_{air}(\kappa)}{1-a_{air}(\kappa)a_{quartz}(\kappa)}\right|^2 \frac{3}{8\pi}\frac{n_{quartz}^2\cos\theta}{n_o^2}\sqrt{n_o^2-\frac{n_o^2}{n_e^2}\kappa^2}\;2\pi\sin\theta d\theta$$

For the TE part of a horizontal dipole we have

(15) $$I_{hor}^{TE}(\theta)2\pi\sin\theta d\theta = P_{vac}(1-r_{quartz}^2(\kappa))\left|\frac{1+a_{air}(\kappa)}{1-a_{air}(\kappa)a_{quartz}(\kappa)}\right|^2 \frac{3}{8\pi}\frac{n_{quartz}^2\cos\theta}{\sqrt{n_o^2-\kappa^2}}2\pi\sin\theta d\theta$$

The experimentally measured intensity profile can then fitted to $I_{hor}^{TM}(\theta)$ and $I_{ver}^{TM}(\theta)$ by simple linear regression to yield the relative weight factors.

(16) $$\sum_i (I_{meas}^{TM}(\theta_i) - w_{hor}I_{hor}^{TM}(\theta_i) - w_{ver}I_{ver}^{TM}(\theta_i))^2 \to \mathrm{Min}$$

where the sum extends over the measurement angles θ_i.

The weights are then given by the linear equation

(17) $$\begin{aligned} I_{ver}\cdot I_{ver}\, w_{ver} + I_{ver}\cdot I_{hor}\, w_{hor} &= I_{ver}\cdot I_{meas} \\ I_{ver}\cdot I_{hor}\, w_{ver} + I_{ver}\cdot I_{ver}\, w_{hor} &= I_{hor}\cdot I_{meas} \end{aligned}$$

with

(18) $$I_\alpha\cdot I_\beta = \sum_i I_\alpha(\theta_i)\cdot I_\beta(\theta_i) \qquad \alpha,\beta\in\{\mathrm{hor},\mathrm{ver},\mathrm{meas}\}$$

Finally the orientation parameter is given by $\rho = \frac{w_{hor}}{w_{hor}+w_{ver}}$.

Using these formulas it is easy to write a program to determine orientation parameters from measured values.

Acknowledgements: The author of this mathematical appendix wishes to thank Ian Zwiers from Lumerical for helpful technical discussions. Commercial software for the simulation of dipole emission in general microcavities is available from Lumerical (www.lumerical.com).

References:

S1 K. Neyts, *Simulation of light emission from thin-film microcavities*, *J. Opt. Soc. Am. A*, 1998, **15**, 962–971.

S2 C.-K. Moon, S.-Y. Kim, J.-H. Lee, J.-J. Kim, *Luminescence from oriented emitting dipoles in a birefringent medium*, *Opt. Express*, 2015, **23**, A279–A291.

S3 L. Penninck, P. De Visschere, J. Beeckman, K. Neyts, *Dipole radiation within one-dimensional anisotropic microcavities: a simulation method*, *Opt. Express*, 2011, **19**, 18558−18576.

S4 J. A. E. Wasey, A. Safonov, I. D. W. Samuel, W. L. Barnes, *Effects of dipole orientation and birefringence on the optical emission from thin films*, *Opt. Commun.*, 2000, **183**, 109−121.

S5 P. C. Clemmow, *The theory of electromagnetic waves in a simple anisotropic medium*, *Proc. I.E.E.*, 1963, **110**, 101−106.

S6 L. Novotny, B. Hecht, *Principles of Nano-Optics*, Cambridge University Press, Cambridge: 2006

S7 P. Yeh, *Optical Waves in Layered Media*, Wiley, New York: 1988

Some useful integrals: (Springer Taschenbuch der Mathematik s.155 0.7)

$$\int_0^n \frac{x\,dx}{\sqrt{n^2 - x^2}} = n \qquad \int_0^n \frac{x^3\,dx}{\sqrt{n^2 - x^2}} = \frac{2}{3}n^3 \qquad \int_0^n x\sqrt{n^2 - x^2} = \frac{1}{3}n^3$$
